# A multimodal dataset for authoring and editing multimedia content: The MAMEM project

**DOI:** 10.1016/j.dib.2017.10.072

**Published:** 2017-11-03

**Authors:** Spiros Nikolopoulos, Panagiotis C. Petrantonakis, Kostas Georgiadis, Fotis Kalaganis, Georgios Liaros, Ioulietta Lazarou, Katerina Adam, Anastasios Papazoglou-Chalikias, Elisavet Chatzilari, Vangelis P. Oikonomou, Chandan Kumar, Raphael Menges, Steffen Staab, Daniel Müller, Korok Sengupta, Sevasti Bostantjopoulou, Zoe Katsarou, Gabi Zeilig, Meir Plotnik, Amihai Gotlieb, Racheli Kizoni, Sofia Fountoukidou, Jaap Ham, Dimitrios Athanasiou, Agnes Mariakaki, Dario Comanducci, Edoardo Sabatini, Walter Nistico, Markus Plank, Ioannis Kompatsiaris

**Affiliations:** a Information Technologies Institute, Centre for Research & Technologies Hellas, Greece; b Aristotle University of Thessaloniki, School of Sciences, Department of Informatics, Greece; c Institute for Web Science and Technologies, University of Koblenz-Landau, Germany; d 3rd University Department of Neurology, Aristotle University of Thessaloniki, Greece; e Department of Neurology, Hippokration General Hospital, Thessaloniki, Greece; f Center of Advanced Technologies in Rehabilitation, Sheba Medical Center, Tel Hashomer, Israel; g Human-Technology Interaction, Eindhoven University of Technology, The Netherlands; h Muscular Dystrophy Association, MDA Hellas, Greece; i EB Neuro S.p.A, Italy; j Senso Motoric Instruments - SMI, Germany; k Department of Physiology and Pharmacology, Sackler Faculty of Medicine, Tel Aviv University, Tel Aviv, Israel; l Sagol School of Neuroscience, Tel Aviv University, Tel Aviv, Israel; m Department of Neurological Rehabilitation, The Chaim Sheba Medical Center at Tel-HaShomer, Tel-HaShomer, Israel; n Department of Occupational Therapy, University of Haifa, Haifa, Israel; o WAIS Research Group, University of Southampton, UK

## Abstract

We present a dataset that combines multimodal biosignals and eye tracking information gathered under a human-computer interaction framework. The dataset was developed in the vein of the MAMEM project that aims to endow people with motor disabilities with the ability to edit and author multimedia content through mental commands and gaze activity. The dataset includes EEG, eye-tracking, and physiological (GSR and Heart rate) signals collected from 34 individuals (18 able-bodied and 16 motor-impaired). Data were collected during the interaction with specifically designed interface for web browsing and multimedia content manipulation and during imaginary movement tasks. The presented dataset will contribute towards the development and evaluation of modern human-computer interaction systems that would foster the integration of people with severe motor impairments back into society.

**Specifications table**

**Table:** 

Subject area	*Human Computer Interaction*
More specific subject area	*Brain-Computer Interfaces, Eye controlled interfaces, Stress detection*
Type of data	*EEG, GSR, HR, eye tracking, and interaction events in Matlab arrays*
How data was acquired	*Electroencephalography, Eye trackers, Galvanic Skin Response and Heart rate sensors*
Data format	*Raw*
Experimental factors	*Archiving*
Experimental features	*This dataset can be used to assess interaction of healthy subjects and patients with motor impairments with computers and assess algorithms for Sensorimotor Rhythms (SMR) and Error Related Potentials (ErrPs) detection in EEG signals.*
Data source location	*Thessaloniki, Athens, Tel Aviv*
Data accessibility	*Data is downloadable without restriction using the following URL:*https://doi.org/10.6084/m9.figshare.5231053

**Value of the data**

• A benchmark dataset for the development and evaluation of novel human computer interaction algorithms.
• The first dataset with multimodal biosignals captured during editing and authoring multimedia content both from healthy and motor impaired individuals.
• A novel dataset combining biosignals and eye tracking information during human computer interaction.

## 1 Data

This work describes the collection and archiving of a large, multimodal and multi-subject dataset gathered in the context of the MAMEM project^1^ (an extensive description of the dataset and the experimental procedures can be found in MAMEM's technical report [1]). MAMEM's goal is to integrate people with motor disabilities back into society by endowing them with the critical skill of managing and authoring multimedia content using novel and more natural interface channels. These channels are controlled by eye-movements and mental commands/brain (expressed through EEG components), significantly increasing the potential for communication and exchange in leisure (e.g., social networks) and non-leisure context (e.g., workplace). In this vein, a large dataset of EEG, GSR, HR, and eye-tracking signals have been recorded under different experimental protocols where 34 subjects (18 healthy and 16 patients) are using a novel, eye-tracking based, web browsing system, namely the GazeTheWeb browser [2], [3]. The dataset was created by gathering signals both from healthy subjects and subjects with motor impairments. The dataset is intended to be exploited by researchers working on HCI and BCI systems’ development and more particularly on multimedia editing and authoring related applications.

## 2 Experimental design, materials and methods

### 2.1 Subjects

In total, 34 subjects took part in the experiments. There were 16 participants with motor impairments [13 male, Age: M(SD)= 47.7 (9.75)], namely, six with Parkinson's disease (PD), six with Neuromuscular disorders (NMD) and four with Spinal Cord Injuries (SCI). Moreover, there were 18 able-bodied participants [13 male, Age: M(SD)= 45 (13)] matched in socio-demographic profile with patients in every cohort separately. In total 26 male and 8 female subjects took part in the study. None of both cohorts of subjects had previously participated in a BCI study. All demographic information along with subjects’ responses in questionnaires concerning the software design and usability are presented in Case Reported Files (CRFs) accompanying the dataset.

### 2.2 Equipment

The signal capturing platform was divided into two different configurations, a “Heavyweight” configuration consisting of high-end devices able to capture high quality signals and a “Lightweight” configuration that is relatively less reliable than the previous but consists of more affordable consumer-based devices. This separation applies only for the EEG recording and eye-tracker devices since the GSR and HR sensor is relevant to both configurations, being both an affordable device and a high quality sensor.

The eye-tracking devices that were selected for use in our study were the *SMI REDn Scientific*^2^ eye tracker for the Heavyweight configuration and the *Visual Interaction myGaze n*^3^ eye tracker for the Lightweight configuration. The main difference between those devices is the maximum supported sampling rate for capturing the eye-gaze, being 60 Hz for the Heavyweight configuration and 30 Hz for the Lightweight version. For the EEG recordings, the *BePlusLTM Bioelectric Signal Amplifier*^4^ was used for the Heavyweight configuration, consisting of 61 EEG electrodes, 1 reference and 1 ground electrode (see Supplementary Material). The reference electrode is placed in the area between FCZ and FZ and the ground between CPZ and PZ. Skin impedance was reduced using a special electro conductive cream and was kept below 10 KΩ for all electrodes. The *Emotiv EPOC*^5^ device was selected for Lightweight configuration, which is equipped with 14 saline-based, wet-contact resistive electrodes, covering the 10–20 electrode area, fixed to flexible plastic arms of a wireless headset. The Shimmer3 GSR+ device was used in both light and heavy-weight configurations for capturing the GSR and HR signals.

### 2.3 Experimental protocol

#### 2.3.1 Training – GazeTheWeb Part

The first part of the experiment involved the engagement of the participants with the GazeTheWeb [2] tool by performing different tasks. Tasks were divided into three main categories (i.e., basic, intermediate and advanced) depending on the difficulty level. *Basic* tasks, aiming on the correct use of the participant's gaze were divided into two sub-categories (Basic 1 and Basic 2). *Intermediate* tasks included browsing and typing activities and the user was instructed to use components such as scroll up/down and copy-paste, but more importantly to use of the virtual keyboard. The *advanced* category focused on the use of the interaction elements provided by the GazeTheWeb Browser.

#### 2.3.2 Error related potentials (ErrPs)

The objective of the ErrPs experiment was to measure the brain activation of each participant while typing words and identify the brain signals of unintentional letter typing. The letters were selected after a dwell time of 0.5 s and then there was a preview of the selected letter by turning its color to white for 1 s. During the preview time, the participant was advised not to gaze away from the selected letter and not to move to the next one immediately since we were trying to capture their brain activations after each letter selection. It was made clear to the participants that erroneous actions should be perceived in a letter-by-letter manner and be corrected as soon as possible since checking for errors (e.g., miss-typed letters) at the end of each word or even the whole sentence would not add any value in our research. Moreover, subjects were asked to refrain their movement in order to avoid artifacts (such as eye blinks, jaw clenched etc.) that contaminate the EEG signal. Five sentences (S1-S5) were shown to the subjects, written clearly with large fonts, in order to be typed using the provided keyboard that operates with an eye-tracker: S1- “My dog barked at the mail carrier”. S2 - “This sentence has only six words”. S3 - “I paid five dollars to buy a ticket”. S4 - “The conference ended at five in the afternoon”. S5 - “The quick brown fox jumps over the lazy dog”. During the experimental procedure, the ongoing typing sentence should be accessible at any time. All sentences should be written using lowercase letters. The end of each sentence was followed by short-time breaks. At the beginning of each session, participants typed their name or some other “foo” phrase in order to familiarize with the keyboard settings.

#### 2.3.3 Sensorimotor rhythms

The SMR experimental protocol consists of two basic steps. At the first step, the EEG data are acquired without showing any visual feedback to the user (calibration step), while, at the second step feedback is incorporated into the overall procedure (feedback step). At the first step (calibration step), the subject tries to imagine the execution of a movement from the left or right hand. Each trial starts with a fixation cross. Some seconds later a visual cue (an arrow pointing either to the left or right) is presented. Afterwards the subjects have to imagine the corresponding hand movement over a period of time, usually 4–6 s (imaginary period). Each trial is followed by a short break. Also, a randomized short period is added to the break in order to avoid subjects adaptation. At the feedback step, feedback is provided to the subject during the imaginary period. Depending on the cue, the subject tries to move accordingly the feedback (usually towards the left or right side) by imagining left or right hand movements, respectively.

**Fig. 1:**
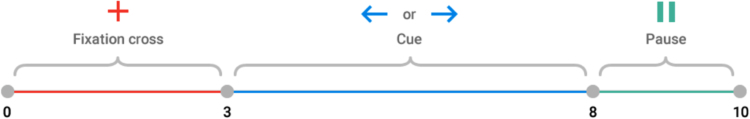
Timing events of a trial during calibration step.

**Fig. 2:**
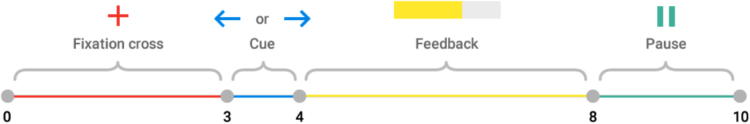
Timing events of a trial during feedback step.

In our analysis, two sessions were recorded for each subject at the same day. In the first session, the subject was instructed to execute the real movement while in the second session to imagine the movement. Each session consists of two parts (calibration and feedback steps). The real movement session was applied only to the participants that were able to move their fingers (i.e., all healthy participants and the patients with Parkinson disease). The calibration step consisted of one run, while the feedback step consisted of 4 runs. In each run 20 trials (10 trials for each class, right or left) were collected. The timing events for a trial belonging to the calibration step are provided in Fig. 1, while the timing events of a trial from the feedback step are shown in Fig. 2.

#### 2.3.4 Dictated Tasks - GazeTheWeb

The Dictated Tasks part was the last part of the experimental protocol and was a simulation of GazeTheWeb (GTW) browser usage in everyday activities. Participants needed to complete four different scenarios (sending an email, editing an image, posting a tweet and watching a YouTube video) composed by several steps that were verbally provided to them by the experimenter. In the first scenario the user ought to reply to an email via Gmail. In order to do so, he/she should sign in using the virtual keyboard with the credentials of an account created for the experiment's purposes. A specific mail from the inbox should be opened and the reply button should be selected. In the email body the phrase “Hello World” should be inserted and hitting the send button would conclude the scenario. The second scenario included the manipulation of an image file with the use of *picresize,*^6^ an online photo editing software. The user was asked to select one of the three sample images to be edited. The next step required the participant to rotate the selected image, followed by the selection of one special filter effect to be applied on the image. The completion step was to select the button “I’m Done, Resize My Picture!”. The third scenario aimed in the use of Twitter. The participant, using an account made for the experiment's purposes, had to search for the account “MAMEM Project” and follow it. Next, a tweet shall be posted. Users were encouraged to create a tweet with few words stating their experience during the experimental procedure. Finally, the use of YouTube was simulated in the last scenario and was initiated with the search of the keyword “gazetheweb mamem”. The next step was to select one of the videos provided by the searching process. Once the video was initiated, it should be paused and played while focusing in the video (like clicking on the video in order to pause/play it). The final step was to close the last opened tab using the appropriate button.

#### 2.3.5 Execution procedure

At the beginning of the experimental procedure, the participant filled in and signed the necessary consent form and was briefed about the incidental findings policy. The heavy-weight system was set up and tested. This step included the initialization of the *Red N (SMI)* eye-tracker, the activation of the GSR/HR sensor and the initialization of all applications that were required for simultaneous signal collection.

The GTW-Game was initialized and the participant filled-in an online form with his credentials to start the game. The participant executed all basic, intermediate and advanced tasks of the GTW-Game. The staff was always available to intervene in case the participant was frustrated. After finishing each group of tasks in the game, the participant filled-in a corresponding task analysis sheet for all task groups.

After the end of the GTW-Game training tasks, the staff saved the generated files. There was a 5 min break and after that, usability questionnaires were filled-in by the participant. The staff proceeded with putting on the heavyweight configuration, and starting all related recording applications. The participant was also briefed about the ErrPs keyboard experiment.

The participant started the new task, using the ErrPs-designed GTW keyboard, and at first the system collected recordings in resting state using a fixation cross for 3 min. After that, the participant was asked by the staff to type a set of predefined sentences (sentences S1-S5). Another 3 min resting state phase initiated and then the ErrPs trial concluded with the staff saving all generated files. Subsequently, the SMR experiment launched, without the use of the eye-tracker. It constituted of a 3 min resting state with a fixation cross, two SMR experiments and another 3 min resting state phase.

After a 15 min break, the Dictated tasks trial initiated. It constituted of social media actions that the participant had to perform. More specifically the actions were (as previously described): i) Sending an e-mail, ii) Editing a photo, iii) Using Twitter to send a tweet, iv) Using YouTube to play/pause a video. The dictated tasks phase ended with the participant filling in the dictated tasks analysis sheet. Finally, another software-design-related questionnaire was filled-in by the participant.

**Fig. 3:**
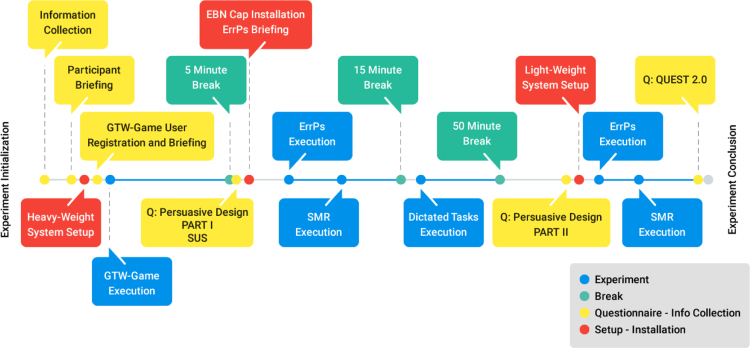
Experimental procedure timeline.

Subsequently, there was a 50 min break and, for a selected set of participants, the second part of the trials commenced. The staff switched the experimental configuration to the lightweight one. More specifically, the *RedN eye-tracker* was replaced with the *myGaze eye-tracker* and the *Emotiv EEG* cap was used. The same ErrP experiments took place. There was also 2 phases of 3-min resting states with a cross fixation as above, at the start and end of the ErrP experiment. The SMR experiment was also performed as above with the *Emotiv EEG* cap. After the end of the lightweight configuration trials, the participant filled in a questionnaire concerning the lightweight configuration. Fig. 3 provides a diagrammatic overview of the execution procedure.

### 2.4 Signals and dataset format

The dataset is divided in three major folders concerning the three experimental sites, i.e., AUTH (Thessaloniki),^7^ MDA (Athens),^8^ and SHEBA (Tel Aviv)^9^ where the experiments took place. Inside each aforementioned folder there are subfolders regarding to each subject separately and, for each subject, matlab array files are provided including the recorded data. The latter files correspond to the four different experimental phases plus two for the lightweight configurations, if available. Specifically, the experimental phases regard to: a) the signals recorded during the training process (GazeTheWeb (GTW) training), b) during the ErrP experiment (Heavy and Light-weight configuration), c) during the SMR experiment (Heavy and Light-weight configuration), and d) during the Dictated tasks. Each matlab array file is named with the following pattern, [SITE]_[SUBJECTID]_[Experiment], where “SITE” can be either AUTH, MDA or SHEBA, “SUBJECTID” refers to the ID of each subject and “Experiment” can be either one of the following; a) GTW, b) Heavy_ERRP, c) Light_ERRP, d) Heavy_SMR, e) Light_SMR, and f) DICTATED.

Inside each matlab array file lies a matlab structure variable. The size of that variable indicates the number of streams that have been recorded during the associated experimental phase. Specifically, six types of streams can be found in each file of the dataset. In particular:

• ***Shimmer_[PORT] stream*** includes the measurements that have been recorded from the Shimmer device.^10^ The data is structured as a 2xN matrix of *N* samples, in which the first channel of data is the HR measurements in beats per minute and the second channel is the GSR measurements in KΩ. Both data are sampled at 256 Hz and are always equally sized. For the HR values the “PPG to HR” algorithm that is available for the Shimmer device was used, which estimates the HR values based on the RR intervals between two successive heart beats, thus the values are updated by the time a new heartbeat is detected by the sensor. The data quality is affected in some cases due to the loss of contact of the sensors. Loss of contact in the GSR sensors usually appears on the signal as large peaks and in some rare cases as negative values. In HR data, loss of contact can be identified by 0 values followed by a relatively large peak in the data. It is important that both of these cases should be taken into account before passing the data into any processing algorithm.
• ***EmotivLSL_EEG stream*** refers to the light-weight EEG device that was used by some participants. The data consists of 14 EEG channels (see Supplementary material, Table 2), sampled at 128 Hz. Impedance levels of the sensors were all reported as OK (green color) by the official *Emotiv* application.
• ***EBNeuro_BePlusLTM_[IP_address] stream*** consists of the EEG data from the heavy-weight device. The data are structured as a 62xN matrix of *N* samples (see Supplementary material Table 1 for the corresponding electrodes). Impedance levels of the electrodes were kept as low as possible and always below 10 KΩ. Due to frequent usage of the cap prior to the experiments, the C3 channel was not working during the experiments that were performed in the sites of AUTH and MDA, so the data in the electrode #25 consists only of noise.
• ***iViewXLSL & myGazeLSL streams*** consist of eye-tracker data that were captured from the heavy and light-weight eye-trackers, respectively. Both streams are identical in structure, including two channels of gaze data, the first being the gaze coordinates in pixels, along the horizontal axis of the screen and the other one on the vertical. A difference between the data from the two streams is the maximum sampling rate between the two eye-trackers, being 60 Hz for the heavy-weight version and 30 Hz for the light-weight version. An important thing to note as well, is that in each site, screens with different resolutions were used, so the eye-tracker will be different in terms of scale (i.e. the data range of *x*, *y* values will be different for each site). In data produced in AUTH site, the screen resolution was 1680 × 1050 pixels, in MDA site 1920 × 1080 pixels and in SHEBA 1366 × 768. In some rare cases, the eye-tracker stopped working unexpectedly during the experiments, thus some data points might be missing.
• ***BrowserOutputStream (Events stream)*** is a stream of text markers that are generated from GazeTheWeb during user interaction. This includes key typing, mouse events, web navigation events and also events that are task-specific. A complete list of the events that are logged can be found in Supplementary material, Table 3.

**Fig. 4:**
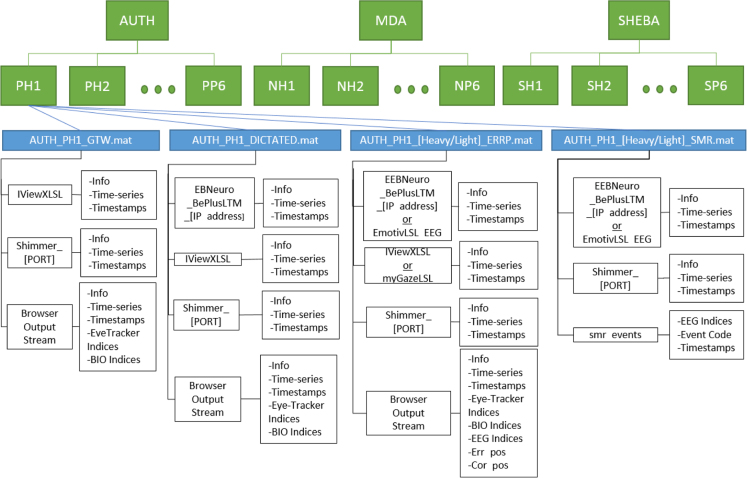
Database structure.

Each of the aforementioned streams is another matlab structure variable with three fields: a) info, b) time_series and c) time_stamps. The “info” field provides several information about the stream, including the name and type of the stream which can be used for identifying it. The “time_series” field includes the actual data of the stream and the equally-sized “time_stamps” field provide the timestamp of each data point. For the case of the BrowserOutputStream there are also additional fields that include the nearest sample indices corresponding to the rest of the streams. For instance, an event “X” will have the following additional fields: a) index of the EEG sample that was recorded at the same timestamp as the event, b) similarly for the eye-tracker stream, and c) similarly for the bio-measurements stream. The abovementioned structure of the dataset is illustrated in Fig. 4.

## Footnotes

1 http://www.mamem.eu/

2 https://www.smivision.com/eye-tracking/product/redn-scientific-eye-tracker/

3 http://www.mygaze.com/products/mygaze-n/

4 http://www.ebneuro.biz/en/neurology/ebneuro/galileo-suite/be-plus-ltm

5 https://www.emotiv.com/epoc/

6 http://www.picresize.com/

7 http://www.med.auth.gr/

8 http://www.mdahellas.gr/

9 https://eng.sheba.co.il/

10 http://www.shimmersensing.com/products/gsr-optical-pulse-development-kit
