# Supplementary material for: A multimodal dataset for authoring and editing multimedia content: The MAMEM project
Source: Data Brief. 2017 Nov 3;15:1048–56. doi: 10.1016/j.dib.2017.10.072 (PMC5709300; doi:10.1016/j.dib.2017.10.072)
Supplement: Supplementary file 1 — Supplementary material [file mmc2.pdf]

Supplementary Material

Heavyweight and Lightweight Channel List

The heavyweight configuration of the EEG system consists of the BePlusLTM Bioelectric Signal Amplifier (64 channels) and an EEG prewired headcap in elastic fabric. The headcap has 61+2 electrodes placed according to the 10-20 system. The electrodes with even numbers are placed on the left hemisphere, whereas the odd ones on the right. A complete list with all electrodes and the corresponding labels of the amplifier are provided on Table A1.

Table 1 Heavyweight channel indexes and electrode names

AF7	1	FC3	17	CP3	33	PO3	49
AF3	2	FC1	18	CP1	34	O1	50
FP1	3	FC2	19	CP2	35	O2	51
FP2	4	FC4	20	CP4	36	PO4	52
AF4	5	FC6	21	CP6	37	PO8	53
AF8	6	FT8	22	TP8	38	OZ	54
F7	7	T3	23	T5	39	AFZ	55
F5	8	C5	24	P5	40	FZ	56
F3	9	C3	25	P3	41	FCZ	57
F1	10	C1	26	P1	42	CZ	58
F2	11	C2	27	P2	43	CPZ	59
F4	12	C4	28	P4	44	PZ	60
F6	13	C6	29	P6	45	POZ	61
F8	14	T4	30	T6	46	SERVICE	62
FT7	15	TP7	31	FPZ	47		
FC5	16	CP5	32	PO7	48		

The lightweight configuration of the EEG system consists of a wireless headcap, connected via bluetooth, with 14+2(reference) electrodes placed according to the ICNS 10-20 system. The electrode numbers correspond the left and right hemisphere the way they do in the heavyweight configuration.

Table 2 Lgithweight Channel list

AF3	1
F7	2

F3	3
FC5	4
T7	5
P7	6
O1	7
O2	8
P8	9
T8	10
FC6	11
F4	12
F8	13
AF4	14

Event List

The complete list of the event markers that have been recorded during our experiments can be found in Table 3 below. These events can be used for marking and segmenting the EEG, eye-tracking, GSR or HR signals. In the first section named "GazeTheWeb - Training & Dictated Tasks", the events that are common for these two tasks are described. Different sections of the table present the events that are unique for the tasks of GazeTheWeb - Training, ErrP experiment and SMR experiment.

Table 3 Events List

Event key	Description
GazeTheWeb - Training & Dictated Tasks	
Click performed	
Close Tab Overview	
Close URL input	
Close Tab Overview	
Display bookmarks	
Edit URL	
GAZE_SELECTED_KEY_[KEY]	Specified which key was pressed
Go back	
Inputting Text: [TEXT]	User typed a text and hit the OK button
Loading URL: [URL]	
Open Tab Overview	
Open bookmark: [BOOKMARK]	
Open new tab	

Submitting Text: [TEXT]	User typed a text and hit the submit button
Text input started	
URL input done	
GazeTheWeb - Training	
page_load__overview	Overview page loaded.
page_load__level_intro	Level intro loaded.
page_load__level_intro_countdown_start	The countdown to start the level has begun.
page_load__level_basic_1_start	BASIC-1 has started.
page_load__level_basic_2_start	BASIC-2 has started.
page_load__level_int_1_start	INT-1 has started.
page_load__level_int_2_start	INT-2 has started.
page_load__level_int_3_start	INT-3 has started.
page_load__level_adv_1_start	ADV-1 has started.
page_load__level_adv_2_start	ADV-2 has started.
page_load__level_adv_3_start	ADV-3 has started.
page_load__level_adv_4_start	ADV-4 has started.
page_load__level_results	Open results page.
page_load__level_failed_text	Openg page that tells user to replay the level.
page_load__level_replay	Initiated level load for replaying.
page_load__next_level	Initiated action to go to next level.
level_basic_1__new_marker	New marker added to BASIC-1
level_basic_1__new_markers_batch	Based on evaluation, another batch of markers was added.
level_basic_2__mole_start	Mole appeared.
level_basic_2__mole_end	Mole dissapeared. Time's up.
level_basic_2__moles_new_batch	Add new batch of moles based on eval.
level_basic_2__mole_hit	Mole HIT.
level_int_1__tower_instance	Got inside the tower.
level_int_1__cave_instance	Got inside the cave.
level_int_2__question_asked_easy	Loaded easy question.
level_int_2__question_asked_med	Loaded medium question.
level_int_2__question_asked_hard	Loaded hard question.
level_int_2__answer_correct	Answered correctly.

level_int_2__answer_wrong	Didn't answer correct.
level_int_3__map_on	Clicked on globe to show the map.
level_int_3__coords_submit	Coordinates submitted.
level_int_3__coords_correct	Coordinates submitted are correct.
level_int_3__coords_wrong	Coordinates submitted are wrong.
level_adv_1__msg_settings	Opened GTW Settings View.
level_adv_1__msg_general	Opened GTW General Settings View.
level_adv_1__msg_gaze_on	Activated Gaze mode.
level_adv_1__msg_gaze_off	Deactivated Gaze mode.
level_adv_1__msg_close	Closed any instance of GTW Interface.
level_adv_2__msg_tabs	Opened GTW Tabs View.
level_adv_2__msg_edit	Started editing a URL.
level_adv_2__msg_keystroke	Keys are pressed on the GTW Keyboard.
level_adv_2__msg_close	Closed any instance of GTW Interface.
level_adv_3__msg_close	Closed any instance of GTW Interface.
level_adv_4__msg_tabs	Opened GTW Tabs View.
level_adv_4__msg_bookmark_add	Added current page as Bookmark.
level_adv_4__msg_new_tab	Added new GTW Tab.
level_adv_4__msg_bookmarks	Accessed the Bookmarks View of GTW.
level_adv_4__msg_open_bookmark	Loaded the saved bookmark.
level_adv_4__msg_tabs_again	Returned to GTW Tabs View.
level_adv_4__msg_tab0	Returned to First tab of GTW.
trophy	Got a trophy.
level_complete	The level is complete.
level_failed	The level has failed.
event__mouse_over	The cursor is on top of an element.
event__mouse_out	The cursor is now out of bounds of the previous element.
event__mouse_down	The hand cursor action (left click) has been fired.
event__browser_back	The BACK button of the browser has been pressed.
event__copy	Pasted text.
SMR Experiment	
101	Start of 1st session (real hand movement)

102	Start of 2nd session (real movement with feedback)
103	Start of 3rd session (imaginary hand movement 1)
104	Start of 4th session (imaginary hand movement 2)
105	Start of 5th session (imaginary hand movement with feedback 1)
106	Start of 6th session (imaginary hand movement with feedback 2)
107	Start of 7th session (imaginary hand movement with feedback 3)
108	Start of 8th session (imaginary hand movement with feedback 4)
201	End of 1st session (real hand movement)
202	End of 2nd session (real movement with feedback)
203	End of 3rd session (imaginary hand movement 1)
204	End of 4th session (imaginary hand movement 2)
205	End of 5th session (imaginary hand movement with feedback 1)
206	End of 6th session (imaginary hand movement with feedback 2)
207	End of 7th session (imaginary hand movement with feedback 3)
208	End of 8th session (imaginary hand movement with feedback 4)
768	Start of Trial
769	Left Arrow presented on the screen
770	Right Arrow presented on the screen
781	Feedback appeared on the screen
786	Cross appeared on the screen
800	End of Trial
32775	Baseline signal started
32776	Baseline signal stopped
ERRP Experiment	
Begin_Rest_1	Start of baseline signal recording 1
Begin_Sentence_1	Begin typing sentence 1
Begin_Sentence_2	Begin typing sentence 2
Begin_Sentence_3	Begin typing sentence 3

Begin_Sentence_4	Begin typing sentence 4
Begin_Sentence_5	Begin typing sentence 5
Begin_Rest_2	Start of baseline signal recording 2
End_Rest_1	End of baseline signal recording 1
End_Sentence_1	End typing sentence 1
End_Sentence_2	End typing sentence 2
End_Sentence_3	End typing sentence 3
End_Sentence_4	End typing sentence 4
End_Sentence_5	End typing sentence 5
End_Rest_2	End of baseline signal recording 2
GAZE_SELECTED_KEY_[KEY]	Specifies which key was typed by the user
Key-Selection-Duration to 0.5 seconds	Key dwell time set to 0.5 seconds (fast mode)
Key-Selection-Duration to 1.0 seconds	Key dwell time set to 1.0 seconds (slow mode)
-1	Typed the wrong character
1	Typed the correct character
